# GWA Analysis for Milk Production Traits in Dairy Sheep and Genetic Support for a QTN Influencing Milk Protein Percentage in the *LALBA* Gene

**DOI:** 10.1371/journal.pone.0047782

**Published:** 2012-10-18

**Authors:** Elsa García-Gámez, Beatriz Gutiérrez-Gil, Goutam Sahana, Juan-Pablo Sánchez, Yolanda Bayón, Juan-José Arranz

**Affiliations:** 1 Departmento Producción Animal, Universidad de León, León, Spain; 2 Department of Molecular Biology and Genetics, Aarhus University, Aarhus, Denmark; 3 Mejora Genética Animal, IRTA, Lleida, Spain; Kansas State University, United States of America

## Abstract

In this study, we used the *Illumina OvineSNP50 BeadChip* to conduct a genome-wide association (GWA) analysis for milk production traits in dairy sheep by analyzing a commercial population of Spanish Churra sheep. The studied population consisted of a total of 1,681 Churra ewes belonging to 16 half-sib families with available records for milk yield (MY), milk protein and fat yields (PY and FY) and milk protein and fat contents (PP and FP). The most significant association identified reached experiment-wise significance for PP and FP and was located on chromosome 3 (OAR3). These results confirm the population-level segregation of a previously reported QTL affecting PP and suggest that this QTL has a significant pleiotropic effect on FP. Further associations were detected at the chromosome-wise significance level on 14 other chromosomal regions. The marker on OAR3 showing the highest significant association was located at the third intron of the *alpha-lactalbumin (LALBA)* gene, which is a functional and positional candidate underlying this association. Sequencing this gene in the 16 Churra rams of the studied resource population identified additional polymorphisms. One out of the 31 polymorphisms identified was located within the coding gene sequence (*LALBA_*g.242T>C) and was predicted to cause an amino acid change in the protein (Val27Ala). Different approaches, including GWA analysis, a combined linkage and linkage disequilibrium study and a concordance test with the QTL segregating status of the sires, were utilized to assess the role of this mutation as a putative QTN for the genetic effects detected on OAR3. Our results strongly support the polymorphism *LALBA_*g.242T>C as the most likely causal mutation of the studied OAR3 QTL affecting PP and FP, although we cannot rule out the possibility that this SNP is in perfect linkage disequilibrium with the true causal polymorphism.

## Introduction

Over the last five years, high-throughput SNP technologies have provided the opportunity to explore the genomes of livestock species to identify regions influencing traits of economic interest. Genome-wide association (GWA) studies are currently replacing traditional QTL linkage mapping analyses as more powerful gene detection tools. In cattle, many GWA studies based on SNP chips have been performed so far for milk production [1], [2], [3], milk protein composition [4], milk fatty acid composition [5], growth [6], [7] and reproductive traits [3], [8], [9], [10], [11]. Moreover, in sheep, the *Illumina OvineSNP50 BeadChip* has previously been used to accomplish medium-density marker genome scans to study categorical or disease-like traits and, in some cases, to identify candidate causative mutations for the traits under examination [12], [13], [14].

However, to date, no GWA analysis related to quantitative traits of economic interest in sheep has been reported, and only the results of classical QTL analysis based on microsatellite markers are available (http://www.animalgenome.org/cgi-bin/QTLdb/OA/index). Despite the close phylogenetic relationship between sheep and cattle, the few QTN previously identified in dairy cattle [15], [16], [17] appear not to have similar effects in dairy sheep, as indicated by analyses performed in Spanish Churra sheep [18]. In dairy cattle, genomic selection is now being successfully implemented based on the existing linkage disequilibrium (LD) between markers and QTL but without relying on the identification of the true causal mutations or QTN [19]. However, the identification of QTN could help to avoid some important limitations of the genomic selection approach, such as the need to genotype large numbers of animals and repeat genotyping after several generations [20]. Gene-assisted selection could be of special interest for applying genomic selection in sheep, in which the limited size of the populations hampers the establishment of training populations and the estimation of marker-QTL effects used to predict breeding values.

In dairy sheep, microsatellite-based whole genome-scans have identified several QTL influencing milk-related traits [21], [22], [23], [24], [25]. In Spanish Churra sheep, only one of the QTL identified for milk traits reached genome-wide significance. This QTL was located on chromosome 3 (OAR3) and influenced milk protein percentage [23]. After an initial increase of microsatellite marker density, some candidate genes, such as *HDAC7* (*Histone deacetylase 7*), *VDR* (*Vitamin D receptor*), *IGFBP6* (*insulin-like growth factor binding protein 6*) and *LALBA* (*alpha-lactalbumin*), were identified within the redefined 13-cM length confidence interval (CI) estimated for this QTL region [26].

In the present work, the *Illumina OvineSNP50 BeadChip* was used to perform a higher density GWA analysis to identify genomic regions influencing milk production traits in Churra sheep. The objectives of this study were, first, to identify, at the population level, novel regions associated with milk production traits that had not previously been identified because of the lower resolution of microsatellite-based scans and/or the limitations of outbred linkage-based studies and, second, to replicate and fine-map QTL previously identified in Churra sheep.

## Materials and Methods

### Resource Population and Phenotypic Data

Blood samples from 1,696 Spanish Churra ewes were collected in order to extract DNA. The ewes were distributed in 16 half-sib families with an average size of 105 daughters per ram (ranging from 29 to 277 animals per half-sib family). The use of animals was performed in compliance with the guidelines approved by the University of Leon ethical commission.

The phenotypes included in the analysis were milk yield (MY), protein yield (PY), fat yield (FY), protein percentage (PP) and fat percentage (FP), which are collected routinely by the National Association of Churra Breeders (ANCHE) through the official milk recording process. The dependent variables used in the association analysis were the yield deviations (YDs) corresponding to the studied traits. The YDs were estimated as averages of the ewes’ raw phenotypic records corrected for fixed environmental effects and the common environmental effect [27]. The calculation of the YDs was performed using multivariate animal repeatability models. The fixed effects used to calculate these YDs were the Herd-Test-Day effect, the birth order, the age of the ewe at parturition (as a covariate nested within birth order), the number of born lambs and the number of weeks of milk production of the ewe.

### SNP Genotyping, Quality Control and Genetic Maps

Genotyping for the *Illumina OvineSNP50 BeadChip* was performed commercially at AROS Applied Biotechnology AS (Aarhus, Denmark) and LABOGENA (Jouy-en-Josas, France). We applied the quality control (QC) criteria previously described by [28] on the raw genotypes: i) GenCall score for raw genotypes >0.15; ii) known location of the marker in the ovine autosomes; iii) call rate per individual >0.9; iv) call rate per SNP≥0.95; v) minor allele frequency (MAF) ≥0.05; vi) correspondence with Hardy-Weinberg equilibrium (HWE) *P*-value >0.00001. After applying these QC criteria, the VerifTyp software was used to impute missing genotypes using pedigree and population information (Boichard D and Druet T, personal communication).

Initially, marker order and positions were based on the Ovine Genome Assembly v2.0 (http://www.livestockgenomics.csiro.au/sheep/oar2.0.php), assuming a conversion ratio of physical to genetic distances of 1 cM to 1 Mb. Next, a control of this initial genetic map was performed using a modified version of CRI-MAP [29], v2.503 (kindly provided by J. F. Maddox). The final genetic map used in the GWA analysis reported here was based on the physical map provided by [28], assuming the conversion ratio indicated above.

### Genome-wide Association Analysis

For the GWA analysis, the following linear mixed model which includes the polygenic effect as a random effect and the genotypes at single SNP markers as fixed effects was applied to the data:
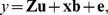
where ***y*** is the vector of phenotypes (YDs) of the animal; ***Z*** is a matrix associating random additive polygenic effects to individuals; ***u*** is a vector containing the random polygenic effects; ***x*** is a vector with genotypic indicator (-1, 0, or 1) associating records to the marker effect; ***b*** is the allele substitution effect determined by the SNP genotype; and ***e*** is the random residual. The random variables ***u*** and ***e*** are assumed to be normally distributed. Specifically, ***u*** is normally distributed with (**0, σ_g_^2^A**), where **σ_g_^2^** is the polygenic genetic variance and **A** is the additive relationship matrix derived from the pedigree. This association analysis was implemented by restricted maximum likelihood (REML) using the DMU software package (available at http://dmu.agrsci.dk), and the marker effect was tested using a Wald test against a null hypothesis of ***b*** = 0.

In a later stage, to examine whether the two most significant SNPs were individually able to explain the QTL effect observed on OAR3 for PP and FP, the above linear mixed model was repeated with the addition of the genotypes of these ‘top’ SNPs as fixed effects in the model, and the analysis was repeated for the rest of the SNPs in this chromosome.

Significance thresholds for the GWA analyses were estimated using a Bonferroni correction for multiple testing. As not all of the markers tested for association were independent because of LD, the Bonferroni correction for the total number of markers in the study is conservative. Therefore, based on the method described by [30], we calculated the number of independently analyzed markers tested for each chromosome and for the entire genome. For the whole genome, the total number of independent SNPs analyzed was 26,965. Hence, the 5% genome-wise significance level corrected for multiple testing corresponded to a nominal *P*-value of 1.85×10^−6^. Finally, the 5% experiment-wise significance threshold was set by accounting for the three independent traits analyzed, which were determined by a principal component analysis performed in R (R Development Core Team, 2008). For simplicity, the statistical significance values given in the text refer to nominal *P-*values.

### Candidate Gene Sequencing Analysis

Sequence analysis was performed for the *LALBA* gene, which was identified as a strong candidate gene for the most significant associations identified in this study. Six primer pairs were designed for the sequence analysis of this gene in the 16 rams of the studied resource population based on the available reference sequences (GenBank Accession No. AB052168; http://www.livestockgenomics.csiro.au/cgi-bin/gbrowse/oarv2.0/). Following the sequencing protocol described by [31], we obtained the forward and reverse strand sequences of the complete *LALBA* gene (4 exons and 3 introns; 1,741 bp), a fraction of the promoter (656 bp) and the 3′UTR region (670 bp) (see Table S1 for the primer sequences and the amplification annealing temperatures for each amplicon). DNA sequence variants (DSVs) in the 16 Churra rams’ sequences were identified using the SeqScape v2.5 software (Applied Biosystems, Foster City, CA). Among all the DSVs identified in this analysis, one of the SNPs located in Exon 1 of the *LALBA* gene (*LALBA*_g.242T>C) was genotyped across the entire population. This genotyping was performed by KBioScience using the fluorescence-based competitive allele-specific PCR (KASPar) assay (for details, see http://www.kbioscience.co.uk).

### Combined Linkage and Linkage Disequilibrium Analysis (LDLA) in OAR3

For the chromosome showing the most significant associations in this study, OAR3, we performed a combined LDLA with the aim of refining the CI of the PP QTL detected on that chromosome [32]. For this purpose, all of the markers on this chromosome from the *Illumina OvineSNP50 BeadChip* and the commercially genotyped *LALBA* SNP were used to perform an LDLA using the QTLmap software [33]. The analysis was performed at every 0.1 cM. The significance thresholds were estimated through a total of 1,000 simulations. To estimate the CIs, the likelihood ratio test statistic (LRT) values were converted into LOD scores, and the LOD drop-off method, as described in [26], was used.

### Concordance Test Performed on the QTL Heterozygous Sires

To assess the possible causality of the *LALBA* gene, all SNPs mapping on OAR3 were phased for the entire population using the PHASEBOOK software [34], following the protocol described by [28]. To identify the heterozygous sires for the OAR3 QTL, we performed a classical regression-based linkage analysis (LA) for the OAR3 markers included in the interval 70–200 Mb using the software GridQTL [35]. Following [36], we performed a fixed position analysis at the closest position to the *LALBA* gene (137 Mb). The families showing a significant QTL effect estimated at the 5% nominal level (ABS(t) >1.96) were considered to be heterozygous (*Qq*) at the QTL. The two phases of the segregating sires were assigned to one of the QTL alleles (*Q* and *q*) according to the sign of the estimated effect for the sire and the daughter’s IBD status as obtained from GridQTL [35]. For the mutations showing concordance with the sires’ QTL status, we then estimated the probability of concordance by chance between the QTL status and the SNP genotype. This probability was calculated as *pA^(NQ)^ x pB^(Nq)^*, where *pA* and *pB* are the allele frequencies of the SNP associated with *Q* and *q* QTL allele, respectively, and *NQ* and *Nq* are the number of concordant *Q* and *q* chromosomes [37].

## Results

**Figure 1:**
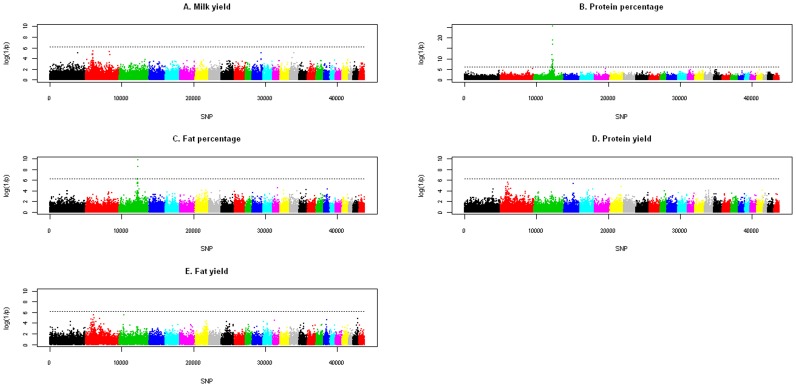
Result from the Genome-wide Association analysis based on the analysis of the *Illumina OvineSNP50 BeadChip.* For the five traits under study (milk yield, MY; protein percentage, PP; fat percentage, FP; protein yield, PY; and fat yield, FY) the log(1/P-value) are depicted here for all the 43,784 SNPs that passed the quality control.

After the QC performed on the raw genotypes, a total of 43,784 SNPs distributed on the 26 ovine autosomes were included in the GWA analysis. Descriptive data for the SNP chip, including the number of SNPs per chromosome, the distance between them, the average MAF and heterozygosity and LD calculations, have been previously reported by [28]. Here, we present the significant associations identified through the GWA analysis for the five milk production traits included in the study (MY, PP, FP, PY and FY). A summary of the discovered experiment- and chromosome-wise significant associations is presented in Table 1. Figure 1 depicts Manhattan plots across the whole genome for the five analyzed traits, with SNP associations represented as log(1/*P*-value) on the y-axis.

**Table 1:** Summary of the significant results obtained from the genome-wide association analysis reported herein.

Significance threshold	Chrom	SNP ID	Position(Mbp)	Trait	Allele substitution effecttrait units (SD units)	*P*-value (Nominal)	*P*-value (Corrected)*
Experiment-wise significant	3	OAR3_147028849	137.3	PP	0.138±0.013 (0.470)	3.78×10^−26^	9.24×10^−23^ (2.77×10^−22^)
	3	OAR3_147028849	137.3	FP	0.169±0.026 (0.297)	1.80×10^−10^	4.39×10^−7^ (1.32×10^−6^)
Chromosome-wise significant	1	OAR1_233634722	216.9	MY	25.824±5.815 (0.210)	9.55×10^−6^	0.030
	2	OAR2_59276935	55.4	MY	26.476±5.766 (0.215)	4.74×10^−6^	0.013
	2	s25113	58.7	PY	−1.467±0.321 (−0.193)	5.27×10^−6^	0.015
	2	OAR2_67323597	63.2	FY	−2.249±0.507 (−0.231)	9.75×10^−6^	0.027
	2	OAR2_182893049	173.1	MY	21.526±4.743 (0.175)	6.10×10^−6^	0.017
	2	OAR2_251955154	239.6	PP	0.051±0.011 (0.173)	5.56×10^−6^	0.015
	3	s40946	35.0	FY	1.811±0.387 (0.186)	3.07×10^−6^	0.008
	4	OAR4_74926053	70.1	PY	−1.552±0.337 (−0.204)	4.46×10^−6^	0.006
	6	DU430803_572	85.4	PP	−0.044±0.009 (−0.148)	4.12×10^−6^	0.005
	7	OAR7_89700312	82.3	PY	−1.562±0.363 (−0.205)	1.78×10^−5^	0.021
	12	OAR12_74759500	68.2	MY	20.021±4.513 (0.163)	9.77×10^−6^	0.009
	14	s66781	13.3	PP	−0.050±0.012 (−0.168)	3.01×10^−5^	0.019
	14	OAR14_15268863	14.9	FY	1.664±0.397 (0.171)	2.87×10^−5^	0.018
	14	OAR14_28957918	27.6	PP	−0.041±0.010 (−0.138)	2.47×10^−5^	0.016
	14	s25830	41.6	FP	0.133±0.032 (0.234)	2.89×10^−5^	0.018
	15	s36641	69.3	PP	0.043±0.010 (0.146)	3.68×10^−5^	0.031
	16	s25440	32.5	MY	−22.771±5.115 (−0.185)	9.10×10^−6^	0.007
	16	OAR16_46325523	43.0	PP	−0.044±0.010 (−0.148)	6.80×10^−6^	0.006
	17	s42157	11.4	PP	−0.055±0.013 (−0.185)	2.66×10^−5^	0.019
	17	OAR17_23761428	21.4	PP	0.067±0.016 (0.227)	1.73×10^−5^	0.013
	17	OAR17_63857104	58.8	FP	−0.079±0.020 (−0.139)	6.03×10^−5^	0.044
	20	OAR20_25029391	23.7	FY	1.977±0.465 (0.203)	2.23×10^−5^	0.013
	20	s69570	29.1	FP	−0.139±0.034 (−0.245)	5.43×10^−5^	0.032
	20	OAR20_32868803	29.6	PP	−0.051±0.013 (−0.173)	5.51×10^−5^	0.032
	23	OAR23_28103191	27.1	PP	−0.042±0.010 (−0.141)	2.08×10^−5^	0.013
	23	s41936	50.8	PY	2.131±0.536 (0.280)	7.33×10^−5^	0.045
	25	s07823	35.3	FY	1.759±0.446 (0.181)	8.25×10^−5^	0.045

* Corrected *P-*values at the experiment-wise or chromosome-wise level are indicated for the associations reaching the corresponding 0.05 significance level. These values were obtained after applying a Bonferroni correction considering the number of independent markers analysed for each chromosome and the entire genome. The 0.05 experiment-wise significance threshold was set by correcting additionally for three independent traits analysed.

The most significant SNPs at each of the significant regions identified at the chromosome-wise and experiment-wise levels are indicated. For each of them, the location chromosome and position (in Megabase pairs), and the nominal and corrected *P*-values are provided. Also included in the table are the magnitude and standard error of the allele substitution effect in both trait units (mL, for yield traits and percentage points, for composition traits) and in phenotypic standard deviations (SD) units (in brackets).

### Experiment-wise Significant Associations

The most significant results from the GWA analysis were obtained for PP on OAR3. The significantly associated SNPs covered a 10.5 Mb-long region of the chromosome (from 130.0 to 140.5 Mb), but the highest LRT values (*P-*value <1.0×10^−10^) were obtained within a 30 Kb interval (from 137.29 to 137.32 Mb). The SNP showing the most significant association was OAR3_147028849 (*P-*value = 3.78×10^−26^), which was located in the third intron of the *LALBA* gene. The same region of OAR3 also showed an experiment-wise significant association with FP (*P-*value = 1.8×10^−10^), in which marker OAR3_147028849 was also identified as the top SNP for this trait. Significant results for FP covered a narrower region (4.3 Mb; from 133.0 to 137.3 Mb) than for PP. The allelic substitution effect of this SNP was positive for both traits, although the magnitude was larger for PP (0.47 SD) than for FP (0.30 SD).

### Chromosome-wise Significant Associations

SNPs on 14 chromosomes showed significant association with the studied traits at the 5% chromosome-wise level. These chromosomes were OAR1, OAR2, OAR3, OAR4, OAR6, OAR7, OAR12, OAR14, OAR15, OAR16, OAR17, OAR20, OAR23 and OAR25. A summary of the significant SNPs associated with the studied traits is presented in Table 1. In general, most of the chromosome-wise significant results were single-point associated SNPs with the exception of a 20-Mb long region on OAR2, which showed significant associations with the three “yield” traits (MY, PY and FY). Significant SNPs in this linkage group were concentrated between 42.7 and 63.2 Mb, although most of them were located in the middle of that region, between 53.0 and 58.0 Mb. The central part of OAR20 exhibited significant associations with more than one trait, although there were also single point associations. At position 23.7 Mb, there was one marker significantly associated with FY, whereas in the region between 29.1 and 29.6 Mb, chromosome-wise significant associations were detected for PP and FP.

### Candidate Gene Sequencing

The sequencing analysis was performed on the 16 rams for the *LALBA* gene, including all exons, introns, the promoter region and 3′UTR of the *LALBA* gene. A total of 3,067 bp were sequenced. The number of DSVs found in the sequenced region was 31, on average one polymorphism at every 98.9 bp. Two of the polymorphisms were one-base indel mutations, and 29 were single-base substitutions (Table S2). Only one of the SNPs identified was located within a translated region of the gene, in Exon 1 (*LALBA_*g.242T>C; GenBank Accession No. AB052168). This mutation was predicted to cause an amino acid substitution of valine to alanine at position 27 of the protein sequence (Val27Ala). The SNPs located in the non-coding region were distributed as follows, 13 mutations were located along the gene introns, including the two indels, whereas eight SNPs were found in the promoter and nine other SNPs in the 3′UTR, respectively.

### Combined Linkage Disequilibrium and Linkage Analysis

**Figure 2:**
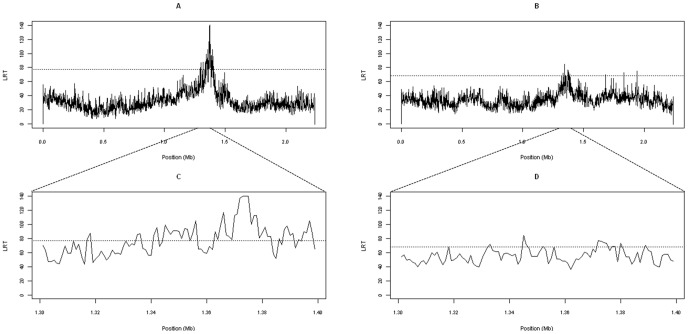
Statistical profiles obtained from the LDLA analyses in OAR3 for milk protein and fat percentages. Together with the LRT profiles obtained across the whole chromosome for protein percentage (PP) (A) and fat percentage (FP) (B), the detailed view of the profile around the *LALBA* gene region (from 1.3 to 1.4 Mb) is also provided for both traits, PP (C) and FP (D).

The results from the LDLA performed in OAR3 for PP and FP are shown in Figure 2. For PP, the most significant result was obtained at 137.3 Mb (LRT = 140.2). According to the significance thresholds set through simulation (LRT = 78.5 for a chromosome-wise *P*-value of 0.0001), the analysis revealed a highly significant QTL located in the middle of a haplotype of 4 SNPs that includes the *LALBA* gene. The order and names of the markers included in this haplotype were *LALBA_*g.242T>C, OAR3_147028849, OAR3_147128672 and OAR3_147275963. Using the LOD drop-off method, the CI covered a 4 Kb-long region from 137.2 Mb to 137.6 Mb in OAR3.

The LDLA results for FP reached chromosome-wise significance, with the maximum LRT value obtained at 134.5 Mb (LRT = 84.6; *P*-value <0.001). The LRT for FP at the location of the PP QTL (137.2 Mb), i.e., the location of LALBA gene, was 76.7 (*P*-value <0.001).

### Genetic Support for the Alpha-lactalbumin (LALBA) Gene

**Figure 3:**
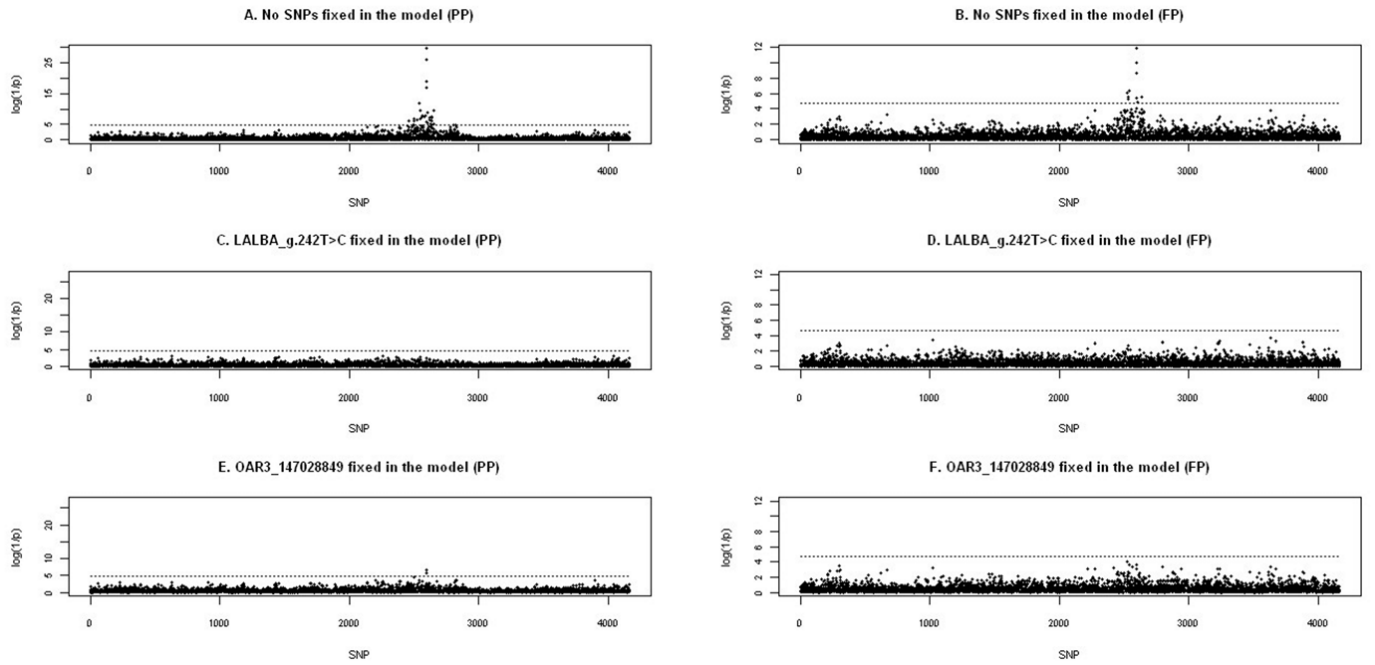
Association analyses for milk protein and fat percentages (PP and FP) including the LALBA_g.242T>C genotypes. Genotypes used in the analysis included SNPs from the *Illumina OvineSNP50 BeadChip* and the single SNP detected in the exonic sequence of the *LALBA* gene (*LALBA*_g.242T>C). The significance thresholds are set at the 5% chromosome-wise level (dashed lines). A and B) Log(1/*P*-value) profiles for the analysis including the pedigree information as a random polygenic effect and each SNPs’ genotypes as a fixed effect in the mixed model for PP and FP, respectively. C and D) Results for the analysis where the genotypes of the *LALBA*_g.242T>C mutation were included as a fixed effect in the mixed model equation for PP and FP, respectively. E and F). Results of the analysis when the SNP OAR3_147028849 was included as a fixed effect in the mixed model equation for PP and FP, respectively.

The association analysis was repeated for OAR3 incorporating the genotyped SNP located in the *LALBA* exon, *LALBA_*g.242T>C, and the results for PP and FP are presented in Figure 3A. For both of the traits, the SNP *LALBA_*g.242T>C showed the most significant association (*P-*value for PP* = *2.34×10^−30^; *P*-value for FP = 1.62×10^−12^). The allele substitution effect was higher for PP (0.46 SD) than for FP (0.30 SD). The second most significantly associated SNP for both PP and FP was also located in the intronic region of the *LALBA* gene that was identified as the top SNP in the initial GWA analysis, OAR3_147028849.

To test the ability of these two SNPs to explain variance in the studied traits, we individually included them in the mixed model equation as fixed factors. When we fixed marker *LALBA_*g.242T>C in the analysis, the significant associations previously identified on OAR3 for PP and FP disappeared (Figures 3C and 3D). Significant associations were also removed when marker OAR3_147028849 was fixed in the FP analysis (Figure 3F). However, when this marker was fixed in the PP mixed model equation significant effects for markers *LALBA*_g.242T>C and OAR3_146832060 still remained (P values = 3.17×10^−7^ and 1.85×10^−6^, respectively). This latter SNP is located 23 Kb upstream from *LALBA* gene (Figure 3E). Using the information provided by the QTLMap LDLA approach, we examined the estimated haplotype effects for PP at the maximum LRT position. We observed that all haplotypes with a positive effect on PP were carrying the *LALBA*_g.242C allele at the *LALBA_*g.242T>C locus, whereas all haplotypes containing the *LALBA*_g.242T allele at that position showed a negative haplotype effect on PP (Table S3).

The LA performed at the fixed position of 137 Mb, the closest position to the *LALBA* gene, showed that three of the sires were segregating for the QTL (ABS(t) >1.96). The concordance test performed using the phases estimated with PHASEBOOK showed that the three sires found to be segregating for the QTL in the LA were heterozygous at the *LALBA*_g.242T>C locus but not at the OAR3_147028849 SNP. We assigned each of the sire’s phases to the *Q* or *q* allele of the QTL according to the daughter’s IBD and the QTL effect. All three sires’ phases carrying the *Q* allele shared the *LALBA*_g.242C allele (Figure S1). Taking into account allelic frequencies at the *LALBA_*g.242T>C locus in the Churra ewes population (*LALBA*_g.242C allele = 0.71 and *LALBA*_g.242T allele = 0.29), the probability of this concordance by chance was estimated to be 0.0087. Within the haplotype block interval considered, from 136.8 to 138.1 Mb, there was another polymorphism, s19950, which showed concordance with the QTL status in the three segregating sires. On the basis of the marker allelic frequencies, the probability of the concordance by chance was 0.0155. In the initial GWA scan for the PP trait, the marker s19950 ranked 9th among the most significant markers (*P*-value = 1.3×10^−9^). The inclusion of this SNP as fixed effect in the model nonetheless yielded highly significant results on OAR3 for both PP (*P*-value = 3.35×10^−22^) and FP (*P*-value = 8.94×10^−9^). Additionally, among the sires that did not reach statistical significance to be considered segregating, there were four sires heterozygous for the *LALBA_*g.242T>C mutation, all of which shared a *LALBA*_g.242C allele at the parental haplotype associated with increased PP. The remaining nine sires of the population were homozygous for the *LALBA*_g.242C allele.

The concordance analysis was also performed for FP. Three sires segregated for the FP QTL at the *LALBA* gene (ABS(t) >1.96). Two of them were coincident with the PP segregating sires, whereas the third, which was homozygous for the *LALBA*_g.242T>C polymorphism, was not segregating for the PP QTL.

## Discussion

In livestock species, GWA analyses have become a powerful strategy to identify DSVs affecting phenotypic variation. This study is, to our knowledge, the first reported GWA study for milk production traits in sheep using a high-throughput SNP array. The analyses reported here provide strong evidence for a highly significant region associated with PP and FP on OAR3 that was previously suggested in classical linkage analyses. Additionally, 14 other chromosome-wise significant associations were detected by the GWA study reported herein.

Among the QTL detected at the chromosome-wise significance level, two regions on OAR2 and OAR20 showed significant associations for more than one trait. In OAR2, we found significant associations for three of the traits under study: MY, PY and FY. Additional within-family linkage analyses showed the same sign for the estimated effects of these QTL, thereby supporting the existence of a pleiotropic QTL that increases milk yield without affecting milk composition. The LOD-drop off CI estimated by our analyses clearly located the QTL in the first third of OAR2 (42.7 to 63.2 Mb). QTLs influencing PP and FP have previously been described in the proximal end of OAR2 in Spanish Churra through a microsatellite-based genome-scan [23], although the estimated CIs from that study spanned most of the chromosome length. The orthologous bovine region corresponding to this OAR2 QTL, on BTA8, has also been associated with milk PY in dairy cattle [38]. According to the Ovine Genome Assembly v2.0, some positional candidate genes located close to the highest associations found in OAR2 (55.39 Mb), within the 48–60 Mb interval, are *TGFBR1* (*transforming growth factor C beta receptor 1*), *IGFBPL1* (*Insulin-like growth factor binding protein-like 1*), *CD72* (*Cytokine 72*), *STOML2* (*stomatin (EPB72)-like 2*), *GalNAc-T12* (*UDP-N-acetyl-alpha-D-galactosamine:polypeptide N-acetylgalactosaminyltransferase 12*), *TLN1* (*talin 1*), *PSAT1* (*phosphoserine aminotransferase 1*), *GCNT1* (*glucosaminyl (N-acetyl) transferase 1 C core 2*) and *RFK* (*riboflavin kinase*). Among them, the *IGFBPL1* gene might be considered a possible functional candidate because its coding protein has a domain that resembles one found in the insulin-like growth factor binding proteins (IGFBPs) that is bound by insulin-like growth factors (IGFs) [39]. These IGFs have been suggested to be associated with milk performance in cattle [40].

Our analysis also detected a region on OAR20 that was associated at the chromosome-wise significance level with three of the studied traits: PP, FP and FY (at 23.7–29.6 Mb). This genomic region includes the major histocompatibility complex class-II genes (MHC-II). Notably, ovine QTL for FP [23] and for MY, FY, PY and FP [21] have been previously identified around the ovine MHC-II region in Spanish Churra sheep and a Sarda×Lacaune backcross population, respectively. Moreover, in the orthologous region of BTA23, QTLs influencing milk production traits have been described [41], with at least one gene located in the MHC-II region (*BoLA-DRB3*) showing a reported association with milk dairy traits [42].

The rest of the chromosome-wise significant associations reported in this paper were, in general, determined by a single, isolated SNP. Although some of these isolated associations could be spurious results or inaccurate chromosomal location mappings resulting from the draft stage of the ovine assembly, it is also possible that they indicate genuine genotype-phenotype associations that would require a more powerful design to be identified. This latter hypothesis is supported, for example, by the observation that the SNP associated with PP at the distal region of OAR6 (DU430803_572, 85.44 Mb) is located within the casein gene cluster (*CSN1S1*, *CSN1S2* and *CSN2*), which encodes the major proteins in sheep milk that determine the technological properties of milk during cheese manufacturing [4]. Genetic variants in these genes have been shown to influence milk casein content [43]. This region on OAR6 has been previously suggested to be associated with milk composition traits in sheep [21], [43], [44]. Another potential functional candidate identified for the chromosome-wise significant associations was found for the FY associated OAR25 peak, which is located in the region surrounding the *NRG3* gene (*Neuregulin 3*). In humans, this gene has been reported to be involved in mammary gland development [45].

The most significant association identified by our GWA analysis has previously been identified in Spanish Churra sheep through classical linkage analyses [23]. Using increased microsatellite markers and a LDLA approach, [26] replicated and redefined the QTL in the same population and postulated the *alpha-lactalbumin* gene (*LALBA*) as a strong functional and positional candidate affecting the traits. Alpha-lactalbumin is a major whey protein that forms a subunit of the lactose synthase binary complex. Because lactose synthase is necessary for the production of lactose and the subsequent movement of water into the mammary secretory vesicles, this enzyme is critical in the lactational control and secretion of milk. Previous studies in *LALBA*-deficient mice have shown the influence of this enzyme on the protein and fat concentration in milk. Homozygous mutant mice produce highly viscous milk that is very rich in fat and protein and devoid of alpha-lactalbumin and lactose [46]. Polymorphisms in the *LALBA* gene were studied in the 1990s as possible markers related to milk production in dairy species. In cattle, one SNP located in the promoter of this gene (ULGR_SNP_U63109_1966) has been found to influence milk traits [4], [47]. This polymorphism was one of the SNPs identified in our population (*LALBA*_g.82G>A), but it was homozygous for all the three sires segregating for the OAR3 QTL in our study. The ovine *LALBA* mRNA sequence reported by [48] (GenBank NM001009797) shows three nucleotide variants regarding the gene sequence used as reference here (GenBank AB052168), one of which corresponds to the coding mutation identified in our population, *LALBA*_g.242T>C. However, to our knowledge, there are no reported studies on the influence of the ovine *LALBA* polymorphisms on milk traits.

The candidacy initially suggested by [26] for the *LALBA* gene was strongly supported by the GWA study reported here. This GWA study identified marker OAR3_147028849, located in the third intron of this gene, as the SNP with the most highly significant association detected on OAR3 for PP and FP. The sequence analysis of the *LALBA* gene in our resource population identified 31 DSVs, one of which was located in the gene-coding region (*LALBA*_g.242T>C), and identified a non-synonymous mutation causing a Val27Ala substitution. To predict the possible structural changes that this amino acid change could cause in the LALBA protein, we used the software PolyPhen (http://genetics.bwh.harvard.edu/pph/index.html). The results indicated that the *LALBA*_g.242T>C mutation does not lead to any structural change in the protein. Moreover, the secondary structure for the protein sequence was identical at the mutation region independent of the *LALBA*_g.242T>C allele [49]. When examining whether the mutation was located in a conserved region of the protein across species, we observed that at the corresponding amino acid position, ruminants and mice have a valine residue, corresponding to the *LALBA*_g.242T allele, whereas other species, such as humans, horse, pig and macaque, have a leucine. The next amino acid of the protein sequence, Phe28Ser, also showed this divergence pattern between species. On the basis of these results, we postulate that the *LALBA*_g.242T allele is the ancestral variant at that position, whereas the *LALBA*_g.242C allele appears to be a more recent mutation, potentially favored by classical selection in Churra sheep because of its favorable effect on milk protein content. Support for the *LALBA*_g.242T allele as the ancestral is also indicated by the large haplotype block shared by the *Q*-carrying chromosomes compared to the discontinuous haplotype blocks of the *q*-carrying chromosomes, as shown in Figure S1.

The results of the different analyses presented here for the *LALBA_*g.242T>C SNP, including the highly significant associations, the cancelation of the QTL effect when it is included as fixed effect in the linear mixed model, and the concordance test, provide strong statistical support that this SNP is the most explanatory QTN accounting for the milk protein and fat content QTL detected in OAR3 in Spanish Churra sheep. The sign of the allelic substitutions effects at the peak QTL position was identical for PP, FP, PY and FY, with slightly higher estimates (in SD units) for the protein quantity traits than for the fat traits (data not shown), whereas the calculated effect for MY was small and negative (-0.05 SD). On the basis of these observations, the high phenotypic correlation between milk content traits (r^2^ = 0.49, based on our YD values) and the significance observed for the PP related QTL, we presume that the target QTL primarily affects PP, with a secondary pleiotropic effect on FP, as previously suggested by [23]. A possible hypothesis supporting this observation is that the Val27Ala substitution at *alpha-lactalbumin* would produce a reduction in the activity of the lactase synthase enzyme with a concomitant reduction in the synthesis of lactose. Because lactose is the major osmoticum in milk and the process of synthesis of lactose is responsible for drawing water into the milk, the reduction of this component would be expected to produce an alteration in the concentration of fat and protein. These two milk components are secreted independently by mammary epithelial cells. All of these observations agree with the concentration effect previously described in *LALBA-*deficient mice for both milk fat and milk protein [46] and support the implication of the *LALBA* gene for the OAR3 QTL effects reported here. Another study on transgenic mice has reported that only PP was significantly affected (*P*-value <0.05) by the dilution effect due to high levels of expression of the *LALBA* gene, whereas minor differences were observed for the rest of milk components, lactose, cream (fat), and total solids [50]. These observations seem to be in agreement with our findings. In addition, the increased frequency of the favorable allele in the Churra population (*f*(*LALBA*_g.242C) = 0.71, *f*(*LALBA*_g.242T) = 0.29) and in the rams (*f*(*LALBA*_g.242C) = 0.78, *f*(*LALBA*_g.242T) = 0.22) may indicate that the Churra selection scheme, for which protein content was one of the first traits to be included as a selection target, has already affected this locus by increasing the frequency of the favorable allele associated with increasing milk protein content, *LALBA*_g.242C. This mutation would be the first ovine-proposed QTN in relation to milk production traits.

It is not an easy task to conclusively prove the identification of a QTN. In addition to genetic analyses providing strong genetic support, identifying a QTN requires validation of the results in different populations and functional supporting assays. In their review, [51] provide a list of conditions for a candidate mutation to meet the burden of proof for QTN and discard possible false positives or neutral polymorphisms in near perfect LD with the causative mutation. In this work, we have demonstrated that the *LALBA*_g.242T>C mutation fulfills, for the PP trait, several of the genetic support-related conditions detailed in that list: (1) it is in the defined CI by linkage and linkage disequilibrium mapping; (2) the gene function is related to the trait; (3) there is concordance between the genotypes and the QTL status in the segregating sires (however, this concordance is not observed for the FP trait); (4) the allele frequency of the allele with favorable effect is higher, possibly because of intensive directional selection, and (5) the detected effect disappears when we fix the candidate SNP genotypes in the linear mixed model equation. Other points listed by [51], including the need to confirm the concordance of the results in additional sheep populations and the performance of functional assays complemented by physiological data will be required to finally prove the causality suggested herein for the *LALBA*_g.242T>C mutation. Moreover, there is a need to confirm whether the same physiological mechanism explains the effects detected on PP and FP, and why some of our analyses were not as conclusive for the FP trait as they are for the PP trait. On this regard, it should be taken into account that in dairy sheep, milk FP shows, in general, a lower heritability than milk PP [52], [53]. Hence, it is possible that the lower heritability for this trait indicates that there are additional non-genetic factors that are not being controlled in the YD estimation model. However, we cannot currently discard this mutation to be in perfect LD with the genuine causal mutation or QTN of the OAR3 QTL reported here. In any case, the *LALBA*_g.242T>C genetic variant appears to be a useful marker for taking advantage of the commercial nature of the population that has served to identify this potential and could be directly used to assist Churra sheep breeders to make informed decisions based on genomic information. The implementation in other dairy sheep populations will depend on the frequency of the variant, the rate of LD with the causal mutation, and the validation of the reported association.

## Supporting Information

Figure S1 **Haplotypes of the heterozygous sires for the PP QTL on OAR3 according to the regression analysis.** The two QTL alleles, *Q* (increased protein content) and *q* (decreased protein content), were assigned to the respective haplotypes, as determined by half-sib family-based regression analysis performed with GridQTL. The genotypes of the 22 markers included in the 136.8–138.1 Mb interval were investigated to check the concordance with the estimated sire’s *QTL* status.
(TIF)

Table S1 **Primers used in the amplification of the ovine** ***alpha-lactalbumin*** **(** ***LALBA*** **) gene.** The size of the six amplified fragments and the melting temperature (T_m_) used in the PCR amplifications are also indicated.
(XLS)

Table S2 **DNA sequence variants detected in the ovine** ***LALBA*** **gene through the sequencing analysis performed on the 16 Churra sires included in this study.** Positions are referred to according to the GeneBank sequence (AB052168).
(XLS)

Table S3 **Description of the haplotypes found at the most significantly associated position in the LDLA QTLmap analysis for protein percentage (PP).** The haplotypes include 4 SNPs: *LALBA*_g.242C>T, OAR3_147028849, OAR3_147128672 and OAR3_147275963. Haplotype frequency, estimated haplotype effect and the precision of the estimate are given in the table.
(XLS)
