# Supplementary figures and images for: GWA Analysis for Milk Production Traits in Dairy Sheep and Genetic Support for a QTN Influencing Milk Protein Percentage in the LALBA Gene
Source: PLoS One. 2012 Oct 18;7(10):e47782. doi: 10.1371/journal.pone.0047782 (PMC3475704; doi:10.1371/journal.pone.0047782)

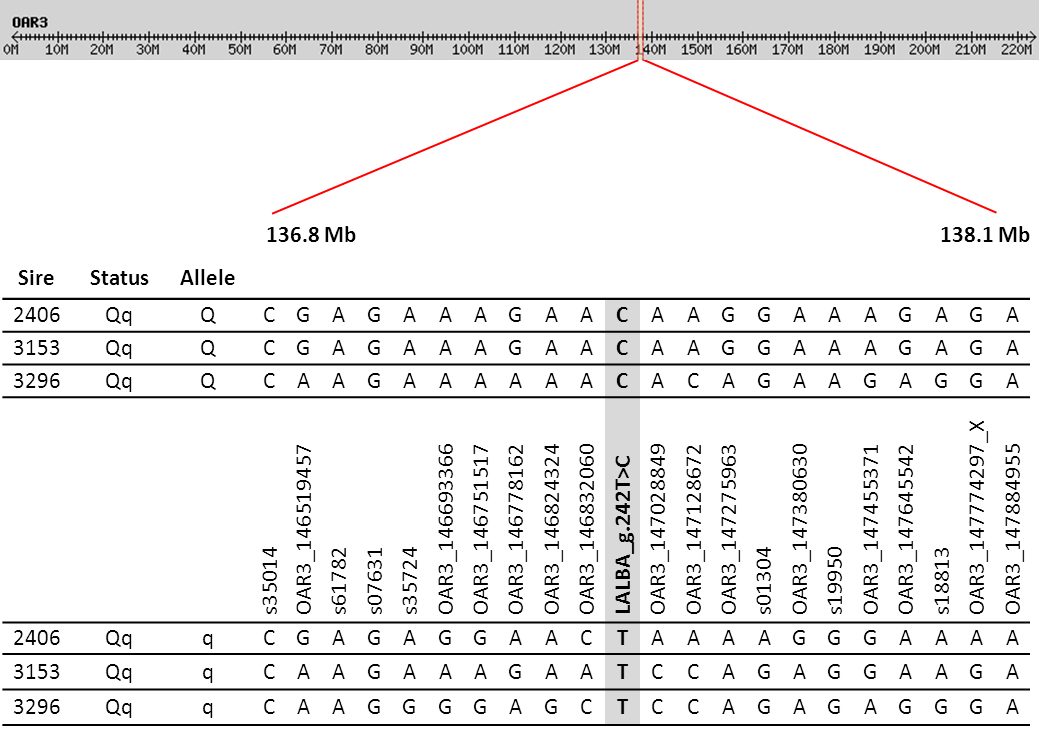

Supplement: Figure S1 — Haplotypes of the heterozygous sires for the PP QTL on OAR3 according to the regression analysis. The two QTL alleles, Q (increased protein content) and q (decreased protein content), were assigned to the respective haplotypes, as determined by half-sib family-based regression analysis performed with GridQTL. The genotypes of the 22 markers included in the 136.8–138.1 Mb interval were investigated to check the concordance with the estimated sire’s QTL status. (TIF) [file pone.0047782.s001.tif]
